# On Sequential Bayesian Inference for Continual Learning

**DOI:** 10.3390/e25060884

**Published:** 2023-05-31

**Authors:** Samuel Kessler, Adam Cobb, Tim G. J. Rudner, Stefan Zohren, Stephen J. Roberts

**Affiliations:** 1 Department of Engineering Science, University of Oxford, Oxford OX2 6ED, UK; zohren@robots.ox.ac.uk (S.Z.); sjrob@robots.ox.ac.uk (S.J.R.); 2 SRI International, Arlington, VA 22209, USA; adam.cobb@sri.com; 3 Department of Computer Science, University of Oxford, Oxford OX1 3QG, UK; tim.rudner@cs.ox.ac.uk

**Keywords:** continual learning, lifelong learning, sequential Bayesian inference, Bayesian deep learning, Bayesian neural networks

## Abstract

Sequential Bayesian inference can be used for *continual learning* to prevent catastrophic forgetting of past tasks and provide an informative prior when learning new tasks. We revisit sequential Bayesian inference and assess whether using the previous task’s posterior as a prior for a new task can prevent catastrophic forgetting in Bayesian neural networks. Our first contribution is to perform sequential Bayesian inference using Hamiltonian Monte Carlo. We propagate the posterior as a prior for new tasks by approximating the posterior via fitting a density estimator on Hamiltonian Monte Carlo samples. We find that this approach fails to prevent catastrophic forgetting, demonstrating the difficulty in performing sequential Bayesian inference in neural networks. From there, we study simple analytical examples of sequential Bayesian inference and CL and highlight the issue of model misspecification, which can lead to sub-optimal continual learning performance despite exact inference. Furthermore, we discuss how task data imbalances can cause forgetting. From these limitations, we argue that we need probabilistic models of the continual learning generative process rather than relying on sequential Bayesian inference over Bayesian neural network weights. Our final contribution is to propose a simple baseline called *Prototypical Bayesian Continual Learning*, which is competitive with the best performing Bayesian continual learning methods on class incremental continual learning computer vision benchmarks.

## 1. Introduction

The goal of continual learning (CL) is to find a predictor that learns to solve a sequence of new tasks without losing the ability to solve previously learned tasks. One key challenge of CL with neural networks (NNs) is that model parameters from previously learned tasks are “overwritten” during gradient-based learning of new tasks, which leads to *catastrophic forgetting* of previously learned abilities [1,2]. One approach to CL hinges on using recursive applications of Bayes’ Theorem, using the weight posterior in a Bayesian neural network (BNN) as the prior for a new task [3]. However, obtaining a full posterior over NN weights is computationally demanding and we often need to resort to approximations, such as the Laplace method [4] or variational inference [5,6] to obtain a neural network weight posterior.

When performing Bayesian CL, sequential Bayesian inference is performed with an approximate BNN posterior, not the true posterior [7,8,9,10,11,12]. If we consider the performance of sequential Bayesian inference with a variational approximation over a BNN weight posterior, then we barely observe an improvement over simply learning new tasks with stochastic gradient descent (SGD). We will develop this statement further in Section 2.2. Therefore, if we had access to the true BNN weight posterior, would this be enough to prevent forgetting by sequential Bayesian inference?

Our contributions in this paper are to revisit Bayesian CL. (1) Experimentally, we perform sequential Bayesian inference using the true Bayesian NN weight posterior. We do this by using the gold standard of Bayesian inference methods, Hamiltonian Monte Carlo (HMC) [13]. We use density estimation over HMC samples and use this approximate posterior density as a prior for the next task within the HMC sampling process. Surprisingly, our HMC method for CL yields no noticeable benefits over an approximate inference method (VCL Nguyen et al. [9]) despite using samples from the true posterior. (2) As a result, we consider a simple analytical example and highlight that exact inference with a misspecified model can still cause forgetting. (3) We show mathematically that under certain assumptions, task data imbalances will cause forgetting in Bayesian NNs. (4) We propose a new probabilistic model for CL and show that by explicitly modeling the generative process of the data, we can achieve good performance, avoiding the need to rely on recursive Bayesian inference over NN weights to prevent forgetting. Our proposed model, *Prototypical Bayesian Continual Learning* (ProtoCL), is conceptually simple, scalable, and competitive with state-of-the-art Bayesian CL methods in the class-incremental learning setting.

## 2. Background

### 2.1. The Continual Learning Problem

*Continual learning* (CL) is a learning setting whereby a model must learn to make predictions over a set of tasks sequentially while maintaining performance across all previously learned tasks. In CL, the model is sequentially shown *T* tasks, denoted $T_{t}$ for t=1,…,T. Each task, $T_{t}$, is comprised of a dataset $D_{t}={\left\{(x_{i},y_{i})\right\}}_{i=1}^{N_{t}}$, which a model needs to learn to make predictions with. More generally, tasks are denoted by distinct tuples comprised of the conditional and marginal data distributions, $\left\{p_{t}\left(y|x\right),p_{t}\left(x\right)\right\}$. After task $T_{t}$, the model will lose access to the training dataset but its performance will be continually evaluated on all tasks $T_{i}$ for i≤t. We decompose predictors as g=h∘f such that $\hat{y}=g\left(x\right)$. We define *f* as an embedding function mapping f:X→Z and *h* as a head mapping to outputs h:Z→Y. Some continual learning methods use a separate head per task ${\left\{h_{i}\right\}}_{i=1}^{T}$, these methods are called multi-headed while those that use one head are called single-headed.

### 2.2. Bayesian Continual Learning

We consider a setting in which task data arrives sequentially at timesteps, t=1,2,…,T. At the first timestep, t=1, that is, for task $T_{1}$, the model receives the first dataset $D_{1}$ and learns the conditional distribution $p(y_{i}|x_{i},\theta )$ for all $\left(x_{i},y_{i}\right)\in D_{1}$ (*i* indexes a datapoint in $D_{1}$). We denote the parameters θ as having a prior distribution p(θ) for $T_{1}$. The posterior predictive distribution for a test point $x_{1}^{*}\in D_{1}$ is hence:

(1) $$\begin{array}{c} p\left(y_{1}^{*}|x_{1}^{*},D_{1}\right)=\int p\left(y_{1}^{*}|x_{1}^{*},\theta \right)p\left(\theta |D_{1}\right)d\theta . \end{array}$$

We note that computing this posterior predictive distribution requires $p(\theta |D_{1})$. For t=2, a CL model is required to fit $p(y_{i}|x_{i},\theta )$ for $\left(x_{i},y_{i}\right)\in D_{1}\cup D_{2}$. The posterior predictive distribution for a new test point $x_{2}^{*}\in D_{1}\cup D_{2}$ point is:

(2) $$\begin{array}{c} p\left(y_{2}^{*}|x_{2}^{*},D_{1},D_{2}\right)=\int p\left(y_{2}^{*}|x_{2}^{*},\theta \right)p\left(\theta |D_{1},D_{2}\right)d\theta . \end{array}$$

The posterior must thus be updated to reflect this new conditional distribution. We can use repeated application of Bayes’ rule to calculate the posterior distributions $p(\theta |D_{1},\ldots ,D_{T})$ as:

(3) $$\begin{array}{cc} p(\theta |D_{1},\ldots ,D_{T-1},D_{T}) & =\frac{p\left(D_{T}|\theta \right)p\left(\theta |D_{1},\ldots ,D_{T-1}\right)}{p(D_{T}|D_{1},\ldots ,D_{T-1})}. \end{array}$$

In the CL setting, we lose access to previous training datasets; however, using repeated applications of Bayes’ rule Equation (3) allows us to sequentially incorporate information from past tasks in the parameters θ. At t=1, we have access to $D_{1}$ and the posterior over parameters is:

(4) $$\begin{array}{cc} \log p(\theta |D_{1}) & =\log p\left(D_{1}|\theta \right)+\log p\left(\theta \right)-\log p\left(D_{1}\right). \end{array}$$

At t=2, we require $p(\theta |D_{1},D_{2})$ to calculate the posterior predictive distribution in Equation (2). However, we have lost access to $D_{1}$. According to Bayes’ rule, the posterior may be written as:

(5) $$\begin{array}{cc} \log p(\theta |D_{1},D_{2})\; & =\log p\left(D_{2}|\theta \right)+\log p\left(\theta |D_{1}\right)-\log p\left(D_{2}|D_{1}\right), \end{array}$$

where we used the conditional independence of $D_{2}$ and $D_{1}$ given θ. We note that the likelihood $p(D_{2}|\theta )$ is only dependent upon the current task dataset, $D_{2}$, and that the prior $p(\theta |D_{1})$ encodes parameter knowledge from the previous task. Hence, we can use the posterior evaluated at *t* as a prior for learning a new task at t+1. From Equation (3), we require that our model with parameters θ is a sufficient statistic of $D_{1}$, i.e., $p\left(D_{2}|\theta ,D_{1}\right)=p\left(D_{2}|\theta \right)$, making the likelihood conditionally independent of $D_{1}$ given θ. This observation motivates the use of high-capacity predictors, such as Bayesian neural networks, that are flexible enough to learn from $D_{1}$.

#### Continual Learning Example: Split-MNIST

For the MNIST dataset [14], we know that if we were to train a BNN we would achieve good performance by inferring the posterior p(θ|D) and integrating out the posterior to infer the posterior predictive over a test point Equation (1). Therefore, if we were to split the dataset MNIST into 5 two-class classification tasks, then we should be able to recursively recover the multi-task posterior $p\left(\theta |D\right)=p\left(\theta |D_{1}\ldots ,D_{5}\right)$ using Equation (3). This problem is called Split-MNIST [15], where the first task involves the classification of the digits {0,1}, the second task classification of the digits {2,3}, and so on.

We can define three different CL settings [16,17,18]. When we allow the CL agent to make predictions with a task identifier τ the scenario is referred to as *task-incremental*. The identifier τ could be used to select different heads Section 2.1, for instance. This scenario is not compatible with sequential Bayesian inference outlined in Equation (3) since no task identifier is required for making predictions. *Domain-incremental* learning is another scenario that does not have access to τ during evaluation and requires the CL agent to perform classification to the same output space for each task; for example, for Split-MNIST the output space is {0,1} for all tasks, so this amounts to classifying between even and odd digits. Domain incremental learning is compatible with sequential Bayesian inference with a Bernoulli likelihood. The third scenario is *class-incremental* learning which also does not have access to τ but the agent needs to classify each example to its corresponding class. For Split-MNIST, for example, the output space is {0,…,9} for each task. Class-incremental learning is compatible with sequential Bayesian inference with a categorical likelihood.

### 2.3. Variational Continual Learning

Variational CL (VCL; Nguyen et al. [9]) simplifies the Bayesian inference problem in Equation (3) into a sequence of approximate Bayesian updates on the distribution over random neural network weights θ. To do so, VCL uses the variational posterior from previous tasks as a prior for new tasks. In this way, learning to solve the first task entails finding a variational distribution $q_{1}\left(\theta |D_{1}\right)$ that maximizes a corresponding variational objective. For the subsequent task, the prior is chosen to be $q_{1}\left(\theta |D_{1}\right)$, and the goal becomes to learn a variational distribution $q_{2}\left(\theta |D_{2}\right)$ that maximizes a corresponding variational objective under this prior. Denoting the recursive posterior inferred from multiple datasets by $q_{t}\left(\theta |D_{1:t}\right)$, we can express the variational CL objective for the *t*-th task as:

(6) $$\begin{array}{cc} L(\theta ,D_{t}) & =D_{\mathrm{KL}}\left[q_{t}\left(\theta \right)\left|\right|q_{t-1}\left(\theta |D_{1:t-1}\right)\right]-E_{q_{t}}\left[\log p\left(D_{t}|\theta \right)\right]. \end{array}$$

**Figure 1:**
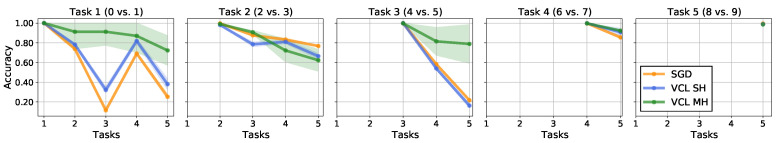
Accuracy on Split-MNIST for various CL methods with a two-layer BNN, all accuracies are an average and standard deviation over 10 runs with different random seeds. We compare an NN trained with SGD (single-headed) with VCL. We consider single-headed (SH) and multi-head (MH) VCL variants.

When applying VCL to the problem of Split-MNIST Figure 1, we can see that single-headed VCL barely performs better than SGD when remembering past tasks. Multi-headed VCL performs better, despite not being a requirement from sequential Bayesian inference Equation (3). Therefore, why does single-head VCL not improve over SGD if we can recursively build up an approximate posterior using Equation (3)? We hypothesize that it could be due to using a variational approximation of the posterior and so we are not actually strictly performing the Bayesian CL process described in Section 2.2. We test this hypothesis in the next section by propagating the true BNN posterior to verify whether we can recursively obtain the true multi-task posterior and so improve on single-head VCL and prevent catastrophic forgetting.

## 3. Bayesian Continual Learning with Hamiltonian Monte Carlo

**Figure 2:**
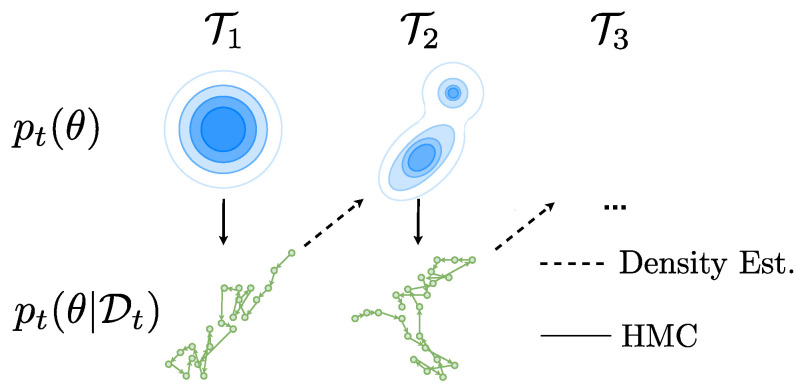
Illustration of the posterior propagation process; priors in blue are in the top row and posterior samples on the bottom row. This is a two-step process where we first perform HMC with an isotropic Gaussian prior for $T_{1}$ then perform density estimation on the HMC samples from the posterior to obtain ${\hat{p}}_{1}\left(\theta |D_{1}\right)$. This posterior can then be used as a prior for the new task $T_{2}$ and so on.

To perform inference over BNN weights we use the HMC algorithm [13]. We then use these samples and learn a density estimator that can be used as a prior for a new task (we considered Sequential Monte Carlo, but it is unable to scale to the dimensions required for the NNs we consider [19]. HMC on the other hand has recently been successfully scaled to relatively small BNNs of the size considered in this paper [20] and ResNet models but at large computational cost [21]). HMC is considered the gold standard in approximate inference and is guaranteed to asymptotically produce samples from the true posterior (in the NeurIPS 2021 Bayesian Deep Learning Competition (https://izmailovpavel.github.io/neurips_bdl_competition), the goal was to find an approximate inference method that is as “close” as possible to the posterior samples from HMC). We use posterior samples of θ from HMC and then fit a density estimator over these samples, to use as a prior for a new task. This allows us to use a multi-modal posterior distribution over θ rather than a diagonal Gaussian variational posterior such as in VCL. More concretely, to propagate the posterior $p(\theta |D_{1})$ we use a density estimator, defined $\hat{p}\left(\theta |D_{1}\right)$, to fit a probability density on HMC samples as a posterior. For the next task $T_{2}$ we can use $\hat{p}\left(\theta |D_{1}\right)$ as a prior for a new HMC sampling chain and so on (see Figure 2). The density estimator priors need to satisfy two key conditions for use within HMC sampling. Firstly, that they are a probability density function. Secondly, that they are differentiable with respect to the input samples.

**Figure 3:**
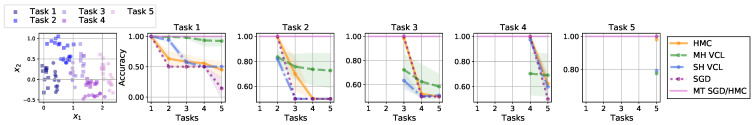
On the left is the toy dataset of 5 distinct 2-way classification tasks that involve classifying circles and squares [22]. Moreover, continual learning binary classification test accuracies over 10 seeds. The pink solid line is a multi-task (MT) baseline accuracy using SGD/HMC with the same model as for the CL experiments.

We use a toy dataset (Figure 3) with two classes and inputs $x\in R^{2}$ [22]. Each task is a binary classification problem where the decision boundary extends from left to right for each new task. We train a two-layer BNN, with a hidden state size of 10. We use Gaussian Mixture Models (GMM) as a density estimator for approximating the posterior with HMC samples. We also tried Normalizing Flows which should be more flexible [23]; however, these did not work robustly for HMC sampling (RealNVP was very sensitive to the choice of random seed, the samples from the learned distribution did not give accurate predictions for the current task and led to numerical instabilities when used as a prior within HMC sampling). To the best of our knowledge, we are the first to incorporate flexible priors into the sampling methods such as HMC.

Training a BNN with HMC on the same multi-task dataset obtains a test accuracy of 1.0. Thus, the final posterior is suitable for continual learning under Equation (3) and we should be able to recursively arrive at the multi-task posterior with our recursive inference method with HMC. The results from Figure 3 demonstrate that using HMC with an approximate multi-modal posterior fails to prevent forgetting and is less effective than using multi-head VCL. In fact, multi-head VCL clearly outperforms HMC, indicating that the source of the knowledge retention is not through the propagation of the posterior but through the task-specific heads. For $T_{2}$, we use $\hat{p}\left(\theta |D_{1}\right)$ instead of $p(\theta |D_{1})$ as a prior and this will bias the HMC sampling for all subsequent tasks. In the next paragraph, we detail the measures taken to ensure that our HMC chains have converged so we are sampling from the true posterior. Moreover, we access the fidelity of the GMM density estimator with respect to the HMC samples. We also repeated these experiments with another toy dataset of five binary classification tasks where we observe similar results Appendix A.

**Figure A4:**
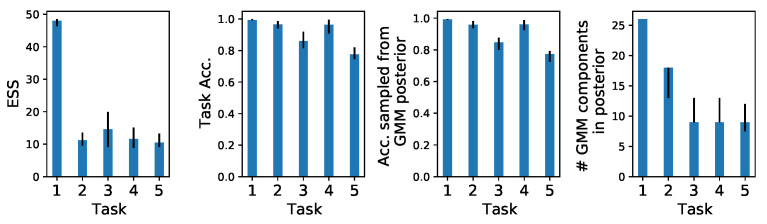
Diagnostics from using a GMM to fit samples of the posterior HMC samples, all results are for 10 random seeds on the toy dataset from Pan et al. [22] (and visualized in Figure 3). **Left**, effective sample sizes (ESS) of the resulting HMC chains of the posterior, all are greater than those reported in other works using HMC for BNNs [20]. **Middle left**. the current task accuracy from HMC sampling. **Middle right**, the accuracy of the BNN when using samples from the GMM density estimator instead of the converged HMC samples. **Right**, The optimal number of components of each GMM posterior fitted with a holdout set of HMC samples by maximizing the likelihood.

**Figure A5:**
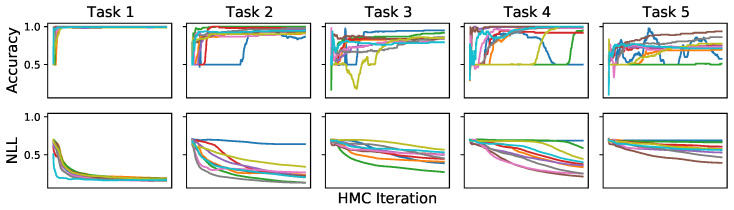
Convergence plots from a randomly sampled HMC chain (of 20) for each task over 10 different seeds for 5 tasks from the toy dataset from [22] (see Figure 3 for a visualization of the data). We use a GMM density estimator as a prior.

For HMC, we ensure that we are sampling from the posterior by assessing chain convergence and effective sample sizes (Figure A5). The effective sample size measures the autocorrelation in the chain. The effective sample sizes for the HMC chains for our BNNs are similar to the literature [20]. Moreover, we ensure that the GMM approximate posterior is multi-modal and has a more complex posterior in comparison to VCL, and that the GMM samples produce equivalent results to HMC samples for the current task (Figure A4). See Appendix B for details.

The 2-d benchmarks we consider in this section are from previous works and are domain-incremental continual learning problems. The domain incremental setting is also simpler [18] than the class-incremental setting and thus a good starting point when attempting to perform exact sequential Bayesian inference. Despite this, we are not able to perform sequential Bayesian inference in BNNs despite using HMC, which is considered the gold standard of Bayesian deep learning. HMC and density estimation with a GMM produces richer, more accurate, and multi-modal posteriors. Despite this, we are still not able to sequentially build up the multi-task posterior or obtain much better results than an isotropic Gaussian posterior such as single-head VCL. The weak point of this method is the density estimation, the GMM removes probability mass over areas of the BNN weight space posterior, which is important for the new task. This demonstrates just how difficult a task it is to model BNN weight posteriors. In the next section, we study a different analytical example of sequential Bayesian inference and look at how model misspecification and task data imbalances can cause forgetting in Bayesian CL.

## 4. Bayesian Continual Learning and Model Misspecification

We now consider a simple analytical example where we can perform the sequential Bayesian inference Equation (3) in closed form using conjugacy. We consider a simple setting where data points arrive online, one after another.

Observations $y_{1},y_{2},\ldots ,y_{t}$ arrive online, and each observation is generated by a hidden variable $\theta_{1},\theta_{2},\ldots ,\theta_{t}∼p$ where *p* is a probability density function. At time *t*, we wish to infer the *filtering distribution*$p(\theta_{t}|y_{1},y_{2},\ldots ,y_{t})$ [24] using sequential Bayesian inference, similarly to the *Kalman filter* [25]. The likelihood is $p\left(y_{t}|\theta_{t}\right)=N\left(y_{t};f\left(\;\cdot \;;\theta_{t}\right),\sigma^{2}\right)$ such that the mean is parameterized by a linear model $y_{t}=f\left(\;\cdot \;;\theta_{t}\right)+\epsilon$ where $\epsilon ∼N(0,\sigma^{2})$ and $f\left(\;\cdot \;;\theta_{t}\right)=\theta_{t}$. We consider a Gaussian prior over the mean parameters θ such that $p\left(\theta_{0}\right)=N\left(\theta_{0};0,\sigma_{0}^{2}\right)$. Since the conjugate prior for the mean is also Gaussian, the prior and posterior are $N(\theta_{t-1};{\hat{\theta }}_{t-1},{\hat{\sigma }}_{t-1}^{2})$ and $N(\theta_{t};{\hat{\theta }}_{t},{\hat{\sigma }}_{t}^{2})$. By using sequential Bayesian inference we can have closed-form update equations for our posterior parameters:

(7) $$\begin{array}{cc} {\hat{\theta }}_{t}={\hat{\sigma }}_{t}^{2}\left(\frac{y_{t}}{\sigma^{2}}+\frac{{\hat{\theta }}_{t-1}}{{\hat{\sigma }}_{t-1}^{2}}\right)={\hat{\sigma }}_{t}^{2}\left(\sum_{i=1}^{t}\frac{y_{i}}{\sigma^{2}}+\frac{{\hat{\theta }}_{0}}{{\hat{\sigma }}_{0}^{2}}\right),\;\;\frac{1}{{\hat{\sigma }}_{t}^{2}} & =\frac{1}{\sigma^{2}}+\frac{1}{{\hat{\sigma }}_{t-1}^{2}}. \end{array}$$

**Figure 4:**
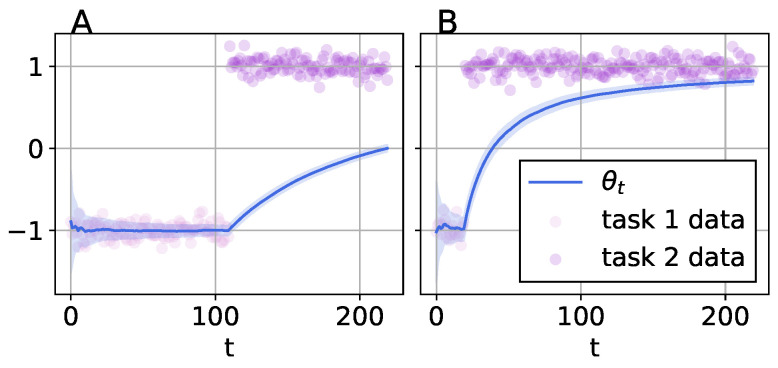
Posterior estimate of the filtering distribution Equation (7) for a linear model where data is divided into two tasks. We study two different scenarios with two tasks or changepoints, with a balanced and an imbalanced task dataset. In scenario **A**, we perform 110 sequential inference updates to the linear model with data from task 1, then another 110 sequential updates with data from task 2. In scenario **B**, the task datasets are imbalanced. We perform 20 sequential Bayesian updates to the linear model with data from task 1 and then 200 updates with data from task 2.

From Equation (7), the posterior mean follows a Gaussian distribution, where the posterior mean is a sum of the online observation and the online prior. Therefore, the posterior mean is a weighted sum of the data and the final value of the posterior is not dependent on the order of the data. We consider the situation where there is a task change (this non-stationarity is referred to as a changepoint in the time-series literature, in Figure 4A at t=110). Concretely, for task 1 the dataset is generated according to $N(-1,\sigma^{2})$, so we want the model to regress to this task. For task 2, the data is generated according to $N(1,\sigma^{2})$ and so we want our continual learning agent to regress well to this task too. As with all continual learning benchmarks, we require our model to retain performance on past tasks and perform equally well on both tasks at the end of training at t=220. From Figure 4A, we can see that the linear model will regress to the first dataset well, as data is seen online and the linear model is updated online. However, as is seen in data from the second task, the linear model eventually tracks the global mean over both tasks Equation (7) rather than a mean for each task Figure 4A. This is even more pronounced when there is a task dataset imbalance Figure 4B.

The model is clearly misspecified since a linear model cannot regress to both of these tasks simultaneously. A more suitable model would be a mixture model, which is able to regress to both task datasets. *Despite performing exact inference, a misspecified model can forget*, Figure 4. Performance on the first task is reduced while learning the second task, this becomes even more pronounced with task dataset imbalances Figure 4B. In the case of HMC, we verified that our Bayesian neural network had a perfect performance on all tasks *beforehand*. In Figure 3, we had a well-specified model but struggled with exact sequential Bayesian inference, Equation (3). When learning with linear models online, we are performing exact inference; however, we have a misspecified model. It is important to disentangle model misspecification and exact inference and highlight that model misspecification is a caveat that has not been highlighted in the CL literature as far as we are aware. Furthermore, we can only ensure that our models are well specified if we have access to data from all tasks a priori. Therefore, in the scenario of *online continual learning* [26,27,28], we cannot know if our model will perform well on all past and future tasks without making assumptions on the task distributions.

## 5. Sequential Bayesian Inference and Imbalanced Task Data

Neural Networks are complex models with a broad hypothesis space and hence are a suitably well-specified model when tackling continual learning problems [29]. However, we struggle to fit the posterior samples from HMC to perform sequential Bayesian inference in Section 3.

**Figure 5:**
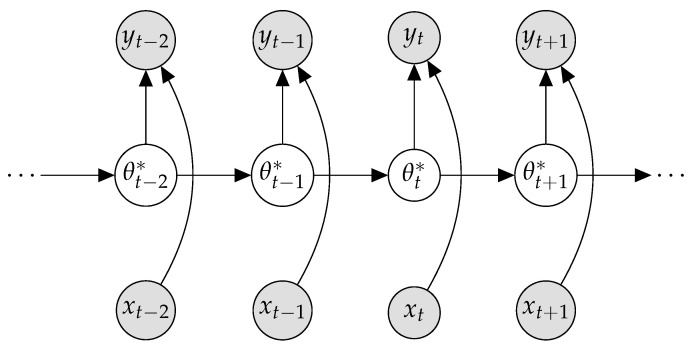
Graphical model for filtering. Grey and white nodes and latent variables are observed, respectively.

We continue to use Bayesian filtering and assume a Bayesian NN where the posterior is Gaussian with a full covariance. By modeling the entire covariance, we enable modeling of how each individual weight varies with respect to all others. We do this by interpreting online learning in Bayesian NNs as filtering [30]. Our treatment is similar to Aitchison [31], who derives an optimizer by leveraging Bayesian filtering. We consider inference in the graphical model depicted in Figure 5. The aim is to infer the optimal BNN weights, $\theta_{t}^{*}$ at *t* given a single observation and the BNN weight prior. The previous BNN weights are used as a prior for inferring the posterior BNN parameters. We consider the online setting, where a single data point $\left(x_{t},y_{t}\right)$ is observed at a time.

Instead of modeling the full covariance, we instead consider each parameter $\theta_{i}$ as a function of all the other parameters $\theta_{-it}$. We also assume that the values of the weights are close to those of the previous timestep [32]. To obtain the updated equations for BNN parameters given a new observation and prior, we make two simplifying assumptions as follows.

**Assumption** **A1.** *For a Bayesian neural network with output $f(x_{t};\theta )$ and likelihood $L(x_{t},y_{t};\theta )$, the derivative evaluated at $\theta_{t}$ is $z_{t}=\partial L\left(x_{t},y_{t};\theta \right){/\partial \theta |}_{\theta =\theta_{t}}$ and the Hessian is H. We assume a quadratic loss for a data point $\left(x_{t},y_{t}\right)$ of the form:*

(8) $$\begin{array}{c} L\left(x_{t},y_{t};\theta \right)=L_{t}\left(\theta \right)=-\frac{1}{2}\theta^{⊤}H\theta +z_{t}^{⊤}\theta , \end{array}$$

*the result of a second-order Taylor expansion. The Hessian is assumed to be constant with respect to $\left(x_{t},y_{t}\right)$ (but not with respect to θ).*

To construct the dynamical equation for θ, consider the gradient for the *i*-th weight while all other parameters are set to their current estimate at the optimal value for the $\theta_{it}^{*}$:

(9) $$\begin{array}{c} \theta_{it}^{*}=-\frac{1}{H_{ii}}H_{-ii}^{⊤}\theta_{-it}, \end{array}$$

since $z_{it}=0$ at a mode. The equation above shows us that the dynamics of the optimal weight $\theta_{it}^{*}$ is dependent on all the other current values of the parameters $\theta_{-it}$. The dynamics of $\theta_{-it}$ are a complex stochastic process dependent on many different variables such as the dataset, model architecture, learning rate schedule, etc.

**Assumption** **A2.** *Since reasoning about the dynamics of $\theta_{-it}$ is intractable, we assume that at the next timestep, the optimal weights are close to the previous timesteps with a discretized Ornstein–Uhlenbeck process for the weights $\theta_{-it}$ with reversion speed $\vartheta \in R_{+}$ and noise variance $\eta_{-i}^{2}$:*

(10) $$\begin{array}{c} p\left(\theta_{-i,t+1}|\theta_{-i,t}\right)=N\left(\left(1-\vartheta \right)\theta_{-it},\eta_{-i}^{2}\right), \end{array}$$

*this implies that the dynamics for the optimal weight are defined by*

(11) $$\begin{array}{c} p\left(\theta_{i,t+1}^{*}|\theta_{i,t}^{*}\right)=N\left(\left(1-\vartheta \right)\theta_{it}^{*},\eta^{2}\right), \end{array}$$

*where $\eta^{2}=\eta_{-i}^{2}H_{-ii}^{⊤}H_{-ii}$.*

In simple terms, in Assumption 2, we assume a parsimonious model of the dynamics, and that the next value of $\theta_{-i,t}$ is close to their previous value according to a Gaussian, similarly to Aitchison [31].

**Lemma** **1.** *Under Assumptions 1 and 2 the dynamics and likelihood are Gaussian. Thus, we are able to infer the posterior distribution over the optimal weights using Bayesian updates and by linearizing the BNN the update equations for the posterior of the mean and variance of the BNN for a new data point are:*

(12) $$\begin{array}{cc} \mu_{t,\mathrm{post}}\; & =\sigma_{t,\mathrm{post}}^{2}\left(\frac{\mu_{t,\mathrm{prior}}}{\sigma_{t,\mathrm{prior}}^{2}\left(\eta^{2}\right)}+\frac{y_{t}}{\sigma^{2}}g\left(x_{t}\right)\right)\;\mathrm{and}\;\frac{1}{\sigma_{t,\mathrm{post}}^{2}}=\frac{g{\left(x_{t}\right)}^{2}}{\sigma^{2}}+\frac{1}{\sigma_{t,\mathrm{prior}}^{2}\left(\eta^{2}\right)}, \end{array}$$

where we drop the notation for the i-th parameter, the posterior is $N(\theta_{t}^{*};\mu_{t,\mathrm{post}},\sigma_{t,\mathrm{post}}^{2})$ and $g\left(x_{t}\right)=\frac{\partial f(x_{t};\theta_{it}^{*})}{\partial \theta_{it}^{*}}$ and $\sigma_{t,\mathrm{prior}}^{2}$ is a function of $\eta^{2}$.

See Appendix E for the derivation of Lemma 1. From Equation (12), we can notice that the posterior mean depends linearly on the prior and a data-dependent term and so will behave similarly to our previous example in Section 4. Under Assumption 1 and Assumption 2, if there is a data imbalance between tasks in Equation (12), then the data-dependent term will dominate the prior term if there is more data for the current task.

In Section 3, we showed that it is very difficult with current machine learning tools to perform sequential Bayesian inference for simple CL problems with small Bayesian NNs. When we disentangle Bayesian inference and model misspecification, we show showed that misspecified models can forget despite exact Bayesian inference. The only way to ensure that our model is well specified is to show that the multi-task posterior produces reasonable posterior predictive distributions p(y|x,D)=∫p(y|x,D,θ)p(θ|D)dθ for one’s application. Additionally, in this section, we have shown that if there is a task dataset size imbalance, then we can obtain forgetting under certain assumptions.

## 6. Related Work

There has been a recent resurgence in the field of CL [33] given the advent of deep learning. Methods that approximate sequential Bayesian inference Equation (3) have been seminal in CL’s revival and have used a diagonal Laplace approximation [3,7]. The diagonal Laplace approximation has been enhanced by modeling covariances between neural network weights in the same layer [8]. Instead of the Laplace approximation, we can use a variational approximation for sequential Bayesian inference, named VCL [9,34]. The variational Gaussian variance of each Bayesian NN parameter can be used to pre-condition the learning rates of each weight and create a mask per task by using pruning [10]. Using richer priors has also been explored [11,35,36,37,38]. For example, one can learn a scaling of the Gaussian NN weight parameters for each task by learning a new variational adaptation parameter which can strengthen the contribution of a specific neuron [39]. The online Laplace approximation can be seen as a special case of VCL where the KL-divergence term Equation (6) is tempered and the temperature tends to 0 [12]. Gaussian processes have also been applied to CL problems leveraging inducing points to retain previous task functions [40,41].

Bayesian methods that regularize weights have not matched up to the performance of experience replay-based CL methods [42] in terms of accuracy on CL image classification benchmarks. Instead of regularizing high-dimensional weight spaces, regularizing task functions is a more direct approach to combat forgetting [43]. Bayesian NN weights can also be generated by a hypernetwork, where the hypernetwork needs only simple CL techniques to prevent forgetting [44]. In particular, one can leverage the duality between the Laplace approximation and Gaussian processes to develop a functional regularization approach to Bayesian CL [45] or using function-space variational inference [46,47].

In the next section, we propose a simple Bayesian continual learning baseline that models the data-generating continual learning process and performs exact sequential Bayesian inference in a low-dimensional embedding space. Previous work has explored modeling the data-generating process by inferring the joint distribution of inputs and targets p(x,y) and learning a generative model to replay data to prevent forgetting [48], and by learning a generative model per class and evaluating the likelihood of the inputs given each class p(x|y) [49].

## 7. Prototypical Bayesian Continual Learning

We have shown that sequential Bayes over NN parameters is very difficult (Section 3), and is only suitable for situations where the multi-task posterior is suitable for all tasks. We now show that a more fruitful approach is to model the full data-generating process of the CL problem and we propose a simple and scalable approach for doing so. In particular, we represent classes by prototypes [50,51] to prevent catastrophic forgetting. We refer to this framework as Prototypical Bayesian Continual Learning, or ProtoCL for short. This approach can be viewed as a probabilistic variant of iCarl [51], which creates embedding functions for different classes that are simply class means and predictions made by nearest neighbors. ProtoCL also bears similarities to the few-shot learning model Probabilistic Clustering for Online Classification [52], developed for few-shot image classification.

**Model.** ProtoCL models the generative CL process. We consider classes j∈{1,…,J}, generated from a categorical distribution with a Dirichlet prior:

(13) $$\begin{array}{c} y_{i,t}∼\mathrm{Cat}\left(p_{1:J}\right),\;p_{1:J}∼\mathrm{Dir}\left(\alpha_{t}\right). \end{array}$$

Images are embedded into an embedding space by an encoder, z=f(x;w) with parameters w. The per class embeddings are Gaussian, and their mean has a prior which is also Gaussian:

(14) $$\begin{array}{c} z_{it}|y_{it}∼N\left({\bar{z}}_{yt},\Sigma_{\epsilon }\right),\;{\bar{z}}_{yt}∼N\left(\mu_{yt},\Lambda_{yt}^{-1}\right). \end{array}$$

**Figure 6:**
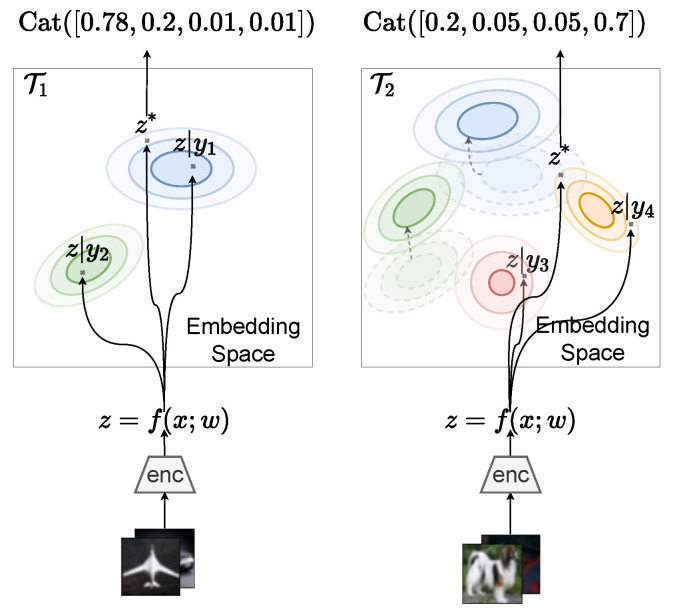
Overview of ProtoCL.

See Figure 6 for an overview of the model. To alleviate forgetting in CL, ProtoCL uses a coreset of past task data to continue to embed past classes distinctly as prototypes. The posterior distribution over class probabilities ${\left\{p_{j}\right\}}_{j=1}^{J}$ and class embeddings ${\left\{{\bar{z}}_{y_{j}}\right\}}_{j=1}^{J}$ is denoted in short hand as p(θ) with parameters $\eta_{t}=\left\{\alpha_{t},\mu_{1:J,t},\Lambda_{1:J,t}^{-1}\right\}$. ProtoCL models each class prototype but does not use task-specific NN parameters or modules such as multi-head VCL. By modeling a probabilistic model over an embedding space, this allows us to use powerful embedding functions f(·;w) without having to parameterize them probabilistically, and so this approach is more scalable than VCL, for instance.

**Inference.** As the Dirichlet prior is conjugate with the categorical distribution and likewise, the Gaussian over prototypes with a Gaussian prior over the prototype mean, we can calculate posteriors in closed form and update the parameters $\eta_{t}$ as new data is observed without using gradient-based updates. We optimize the model by maximizing the posterior predictive distribution and use a softmax over class probabilities to perform predictions. We perform gradient-based learning of the NN embedding function f(·;w) and update the parameters $\eta_{t}$ at each iteration of gradient descent as well, see Algorithm 1.
**Algorithm 1** ProtoCL continual learning1:**Input:** task datasets $T_{1:T}$, initialize embedding function: f(·;w), coreset: M=∅.2:**for** $T_{1}$ **to** $T_{T}$ **do**3:   **for** each batch in $T_{i}\cup M$ **do**4:     Optimize f(·;w) by maximizing the posterior predictive p(z,y) Equation (18)5:     Obtain posterior over θ by updating η, Equations (15)–(17).6:   **end for**7:   Add random subset from $T_{i}$ to M.8:**end for**

**Sequential updates.** We can obtain our parameter updates for the Dirichlet posterior by Categorical-Dirichlet conjugacy:

(15) $$\begin{array}{c} \alpha_{t+1,j}=\alpha_{t,j}+\sum_{i=1}^{N_{t}}I\left(y_{t}^{i}=j\right), \end{array}$$

where $N_{t}$ are the number of points seen during the update at timestep *t*. Moreover, due to Gaussian-Gaussian conjugacy, the posterior for the Gaussian prototypes is governed by:

(16) $$\begin{array}{cc} \Lambda_{y_{t+1}} & =\Lambda_{y_{t}}+N_{y}\Sigma_{\epsilon }^{-1} \end{array}$$

(17) $$\begin{array}{cc} \Lambda_{y_{t+1}}\mu_{y_{t+1}} & =N_{y}\Sigma_{\epsilon }^{-1}{\bar{z}}_{y_{t}}+\Lambda_{y_{t}}\mu_{y_{t}},\;\forall y_{t}\in C_{t}, \end{array}$$

where $N_{y}$ are the number of samples of class *y* and ${\bar{z}}_{y_{t}}=\left(1/N_{y}\right)\sum_{i=1}^{N_{y}}z_{yi}$, see Appendix D.2 for the detailed derivation.

**Objective.** We optimize the posterior predictive distribution of the prototypes and classes:

(18) $$\begin{array}{cc} p(z,y) & =\int p\left(z,y|\theta_{t};\eta_{t}\right)p\left(\theta_{t};\eta_{t}\right)d\theta_{t}=p\left(y\right)\prod_{i=1}^{N_{t}}N\left(z_{it}|y_{it};\mu_{y_{t},t},\Sigma_{\epsilon }+\Lambda_{y_{t},t}^{-1}\right). \end{array}$$

where the $p\left(y\right)=\alpha_{y}/\sum_{j=1}^{J}\alpha_{j}$, see Appendix D.3 for the detailed derivation. This objective can then be optimized using gradient-based optimization for learning the prototype embedding function z=f(x;w).

**Predictions.** To make a prediction for a test point $x^{*}$, the class with the maximum (log)-posterior predictive is chosen, where the posterior predictive is:

(19) $$\begin{array}{cc} p(y^{*}=j|x^{*},x_{1:t},y_{1:t}) & =p\left(y^{*}=j|z^{*},\theta_{t}\right)=\frac{p(y^{*}=j,z^{*}|\theta_{t})}{\sum_{i}p\left(y=i,z^{*}|\theta_{t}\right)}, \end{array}$$

see Appendix D.4 for further details.

**Preventing forgetting.** As we wish to retain the class prototypes, we make use of coresets: experience from previous tasks. At the end of learning a task $T_{t}$, we retain a subset $M_{t}\subset D_{t}$ and augment each new task dataset to ensure that posterior parameters $\eta_{t}$ and prototypes are able to retain previous task information.

**Class-incremental learning.** In this CL setting, we do not tell the CL agent which task it is being evaluated on with a task identifier τ. Therefore, we cannot use the task identifier to select a specific head to use for classifying a test point. Moreover, we require the CL agent to identify each class, {0,…,9} for Split-MNIST and Split-CIFAR10, and not just {0,1} as in domain-incremental learning. Class-incremental learning is more general, realistic, and harder a problem setting, and thus important to focus on rather than other settings, despite domain-incremental learning also being compatible with sequential Bayesian inference as described in Equation (3).

**Implementation.** For Split-MNIST and Split-FMNIST, the baselines and ProtoCL all use two-layer NNs with a hidden state size of 200. For Split-CIFAR10 and Split-CIFAR100, the baselines and ProtoCL use a four-layer convolution neural network with two fully connected layers of size 512 similarly to Pan et al. [22]. For ProtoCL and all baselines that rely on replay, we fix the size of the coreset to 200 points per task. For all ProtoCL models, we allow the prior Dirichlet parameters to be learned and set their initial value to 0.7 found by a random search over MNIST with ProtoCL. An important hyperparameter for ProtoCL is the embedding dimension of the Gaussian prototypes for Split-MNIST and Split-FMNIST, this was set to 128, while for the larger vision datasets, this was set to 32 found using grid-search.

**Figure A6:**
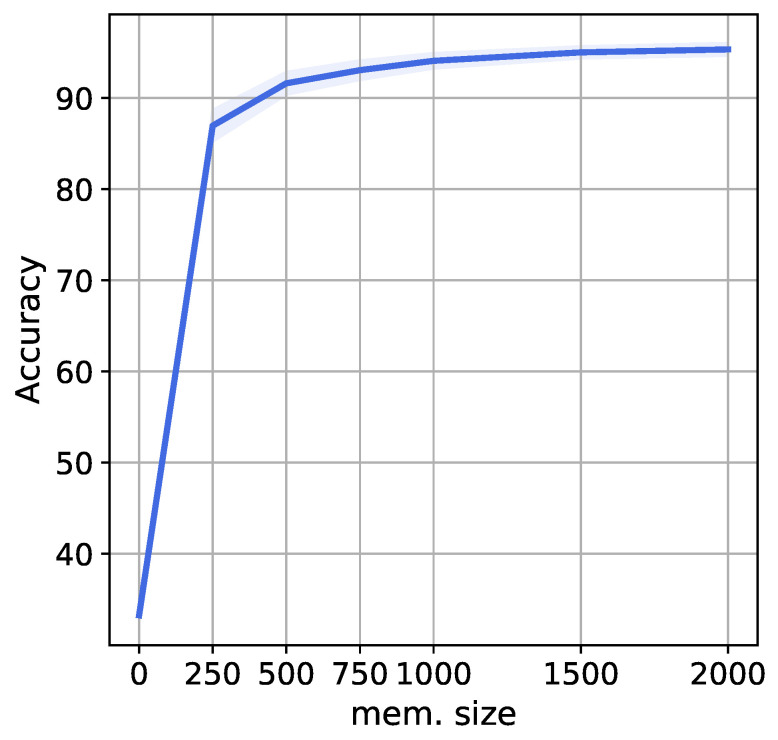
Split-MNIST average test accuracy over five tasks for different memory sizes. On the x-axis, we show the size of the entire memory buffer shared by all five tasks. Accuracies are over a mean and standard deviation over five different runs with different random seeds.

**Results.** ProtoCL produces good results on CL benchmarks on par or better than S-FSVI [47], which is state-of-the-art among Bayesian CL methods while being a lot more efficient to train and without requiring expensive variational inference. ProtoCL can flexibly scale to larger CL vision benchmarks, producing better results than S-FSVI. The code to reproduce all experiments can be found here https://github.com/skezle/bayes_cl_posterior. All our experiments are in the more realistic class incremental learning setting, which is a harder setting than those reported in most CL papers, so the results in Table 1 are lower for certain baselines than in the respective papers. We use 200 data points per task, see Figure A6 for a sensitivity analysis of the performance over the Split-MNIST benchmark as a function of core size for ProtoCL. In Table 2 we show how ProtoCL is able to scale to larger and more challenging CL vision benchmarks. ProtoCL demonstrates competitive performance versus the baselines we consider while at the same time requiring a fraction of the computational cost in terms of training times benchmarked on the same GPU.

The stated aim of ProtoCL is not to provide a novel state-of-the-art method for CL, but rather to propose a simple baseline that takes an alternative route than weight-space sequential Bayesian inference. We can achieve strong results that mitigate forgetting, namely by modeling the generative CL process and using sequential Bayesian inference over a few parameters in the class prototype embedding space. We argue that modeling the generative CL process is a fruitful direction for further research rather than attempting sequential Bayesian inference over the weights of a BNN. ProtoCL scales to 10 tasks of Split-CIFAR100, which, to the best of our knowledge, is the highest number of tasks and classes that have been considered by previous Bayesian continual learning methods.

## 8. Discussion and Conclusions

In this paper, we revisited the use of sequential Bayesian inference for CL. We can use sequential Bayes to recursively build up the multi-task posterior Equation (3). Previous methods have relied on approximate inference and see little benefit over SGD. We test the hypothesis of whether this poor performance is due to the approximate inference scheme by using HMC in two simple CL problems. HMC asymptotically samples from the true posterior, and we use a density estimator over HMC samples to use as a prior for a new task within the HMC sampling process. This density is multi-modal and accurate with respect to the current task but is not able to improve over using an approximate posterior. This demonstrates just how challenging it is to work with BNN weight posteriors. The source of error comes from the density estimation step. We then look at an analytical example of sequential Bayesian inference where we perform exact inference; however, due to model misspecification, we observe forgetting. The only way to ensure a well-specified model is to assess the multi-task performance over all tasks a priori. This might not be possible in online CL settings. We then model an analytical example over Bayesian NNs and, under certain assumptions, show that if there are task data imbalances then this will cause forgetting. Because of these results, we argue against performing weight space sequential Bayesian inference and instead model the generative CL problem. We introduce a simple baseline called ProtoCL. ProtoCL does not require complex variational optimization and achieves competitive results to the state-of-the-art in the realistic setting of class incremental learning.

This conclusion should not be a surprise since the latest Bayesian CL papers have all relied on multi-head architectures or inducing points/coresets to prevent forgetting, rather than better weight-space inference schemes. Our observations are in line with recent theory from [53], which states that optimal CL requires perfect memory. Although the results were shown with deterministic NNs the same results follow for BNN with a single set of parameters. Future research directions include enabling coresets of task data to efficiently and accurately approximate the posterior of a BNN to remember previous tasks.

## Figures and Tables

**Table 1:** Mean accuracies across all tasks over CL vision benchmarks for *class incremental learning* [17]. All results are averages and standard errors over 10 seeds. ${}^{*}$ Uses the predictive entropy to make a decision about which head for class incremental learning.

Method	Coreset	Split-MNIST	Split-FMNIST
VCL [9]	✗	33.01±0.08	32.77±1.25
+ coreset	✓	52.98±18.56	61.12±16.96
HIBNN ${}^{*}$ [11]	✗	85.50±3.20	43.70±20.21
FROMP [22]	✓	84.40±0.00	68.54±0.00
S-FSVI [47]	✓	92.94±0.17	80.55±0.41
ProtoCL (ours)	✓	93.73±1.05	82.73±1.70

**Table 2:** Mean accuracies across all tasks over CL vision benchmarks for *class incremental learning* [17]. All results are averages and standard errors over 10 seeds. Training times have been benchmarked using an Nvidia RTX3090 GPU.

Method	Training Time (s) (↓)	Split CIFAR-10 (Acc) (↑)
FROMP [22]	1425±28	48.92±10.86
S-FSVI [47]	44,434±91	50.85±3.87
ProtoCL (ours)	384±6	55.81±2.10
		**Split CIFAR-100 (Acc) (↑)**
S-FSVI [47]	37,355±1135	20.04±2.37
ProtoCL (ours)	1425±28	23.96±1.34

**Figure A1:**
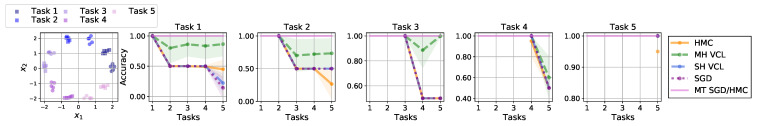
Continual learning binary classification accuracies from the toy Gaussian dataset similar to [44] using 10 random seeds. The pink solid line is a multi-task (MT) baseline test accuracy using SGD/HMC.

**Figure A2:**
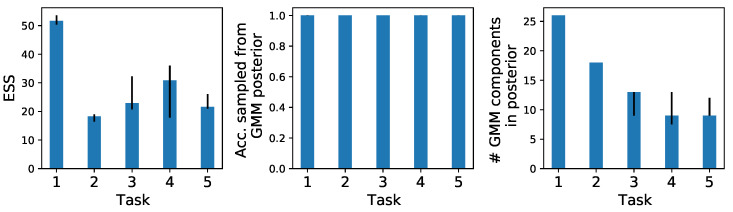
Diagnostics from using a GMM prior fit to samples of the posterior generated from HMC, all results are for 10 random seeds. **Left**, effective sample sizes (ESS) of the resulting HMC chains of the posterior, all are greater than those reported in other works using HMC for BNNs [20]. **Middle**, the accuracy of the BNN when using samples from the GMM density estimator instead of the samples from HMC. **Right**, The optimal number of components of each GMM posterior fitted with a holdout set of HMC samples by maximizing the likelihood.

**Figure A3:**
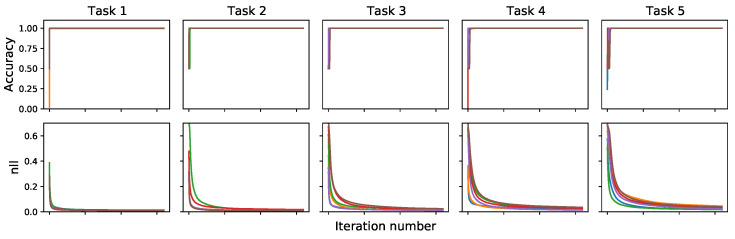
Convergence plots from a one randomly sampled HMC chain (of 20) for each task over 10 different runs (seeds) for 5 tasks from the toy Gaussian dataset similar to Henning et al. [44] (visualized in Figure A1). We use a GMM density estimator as the prior conditioned on the previous task data.

**Figure A7:**
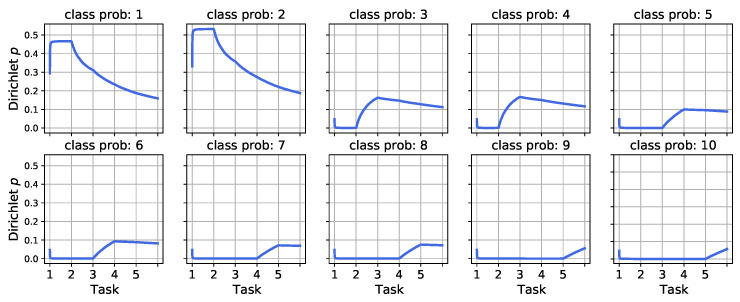
The evolution of the Dirichlet parameters $\alpha_{t}$ for each class in Split-MNIST tasks for ProtoCL. All $\alpha_{j}$ are shown over 10 seeds with ±1 standard error. By the end of training, all classes are roughly equally likely, as we have trained on equal amounts of all classes.

**Figure A8:**
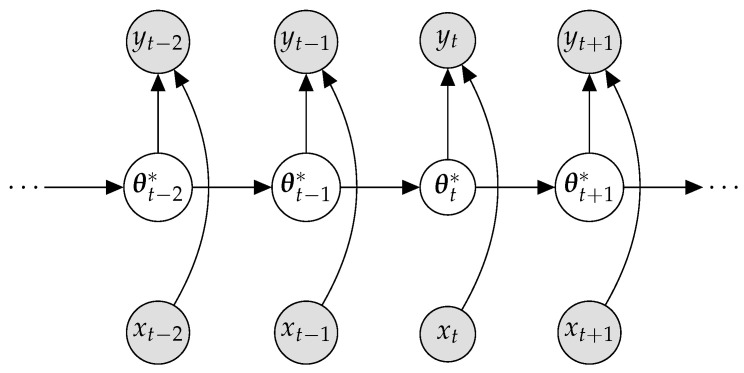
Graphical model of under which we perform inference in Section 5. Grey nodes are observed and white are latent variables.

## Data Availability

All data is publically available, code to reproduce all experiments can be found here https://github.com/skezle/bayes_cl_posterior.

## Abbreviations

The following abbreviations are used in this manuscript:

CL	Continual Learning
NN	Neural Network
BNN	Bayesian Neural Network
HMC	Hamiltonian Monte Carlo
VCL	Variational Continual Learning
SGD	Stochastic Gradient Descent
SH	Single Head
MH	Multi-head
GMM	Gaussian Mixture Model
ProtoCL	Prototypical Bayesian Continual Learning

## Appendix A. The Toy Gaussians Dataset

See Figure A1 for a visualization of the toy Gaussians dataset, which we use as a simple CL problem. This is used for evaluating our method for propagating the true posterior by using HMC for posterior inference and then using a density estimator on HMC samples as a prior for a new task. We construct 5, 2-way classification problems for CL. Each 2-way task involves classifying adjacent circles and squares Figure A1. With a 2 layer network with 10 neurons we obtain a test accuracy of 1.0 for the multi-task learning of all 5 tasks together. Hence, according to Equation (3) a BNN with the same size should be able to learn all 5 binary classification tasks continually by sequentially building up the posterior.

## Appendix B. HMC Implementation Details

We set the prior for $T_{1}$, to $p_{1}\left(\theta \right)=N\left(0,\tau^{-1}I\right)$ with τ=10. We burn-in the HMC chain for 1000 steps and sample for 10,000 more steps and run 20 different chains to obtain samples from our posterior, which we then pass to our density estimator. We use a step size of 0.001 and trajectory length of L=20, see Appendix C for further implementation details of the density estimation procedure. For the GMM, we optimize for the number of components by using a holdout set of HMC samples.

## Appendix C. Density Estimation Diagnostics

We provide plots to show that the HMC chains indeed sample from the posterior have converged in Figure A3 and Figure A5. We run 20 HMC sampling chains and randomly select one chain to plot for each seed (of 10). We run HMC over 10 seeds and aggregate the results Figure 3 and Figure A1. The posteriors $p(\theta |D_{1}),\ldots$ are approximated with a GMM and used as a prior for the second task and so forth.

We provide empirical evidence to show that the density estimators have fit to HMC samples of the posterior in Figure A2 and Figure A4, where we show the number of components of the GMM density estimator, which we use as a prior for a new task, are all multi-modal posteriors. We show the BNN accuracy when sampling BNN weights from our GMM all recover the accuracy of the converged HMC samples. The effective sample size (ESS) of the 20 chains is a measure of how correlated the samples are (higher is better). The reported ESS values for our experiments are in line with previous work which uses HMC for BNN inference [20].

## Appendix D. Prototypical Bayesian Continual Learning

ProtoCL models the generative process of CL where new tasks are comprised of new classes j∈{1,…,J} of a total of *J* and can be modeled by using a categorical distribution with a Dirichlet prior:

(A1) $$\begin{array}{c} y_{i,t}∼\mathrm{Cat}\left(p_{1:J}\right),\;p_{1:J}∼\mathrm{Dir}\left(\alpha_{t}\right). \end{array}$$

We learn a joint embedding space for our data with a NN, z=f(x;w) with parameters w. The embedding space for each class is Gaussian whose mean has a prior which is also Gaussian:

(A2) $$\begin{array}{c} z_{it}|y_{it}∼N\left({\bar{z}}_{yt},\Sigma_{\epsilon }\right),\;{\bar{z}}_{yt}∼N\left(\mu_{yt},\Lambda_{yt}^{-1}\right). \end{array}$$

By ensuring that we have an embedding per class and using a memory of past data, we ensure that the embedding does not drift. The posterior parameters are $\eta_{t}=\left\{\alpha_{t},\mu_{1:J,t},\Lambda_{1:J,t}^{-1}\right\}$.

### Appendix D.1. Inference

As the Dirichlet prior is conjugate with the categorical distribution and so is the Gaussian distribution with a Gaussian prior over the mean of the embedding, then we can calculate posteriors in closed form and update our parameters as we see new data online without using gradient-based updates. We perform gradient-based learning of the NN embedding function f(·;w) with parameters w. We optimize the model by maximizing the log-predictive posterior of the data and use the softmax over class probabilities to perform predictions. The posterior over class probabilities ${\left\{p_{j}\right\}}_{j=1}^{J}$ and class embeddings ${\left\{{\bar{z}}_{y_{j}}\right\}}_{j=1}^{J}$ is denoted as p(θ) for short hand and has parameters are $\eta_{t}=\left\{\alpha_{t},\mu_{1:J,t},\Lambda_{1:J,t}^{-1}\right\}$ are updated in closed form at each iteration of gradient descent.

### Appendix D.2. Sequential Updates

We can obtain our posterior:

(A3) $$\begin{array}{cc} p(\theta_{t}|D_{t}) & \propto p\left(D_{t}|\theta_{t}\right)p\left(\theta_{t}\right) \end{array}$$

(A4) $$\begin{array}{cc} & =\prod_{i=1}^{N_{t}}p\left(z_{t}^{i}|y_{t}^{i};{\bar{z}}_{y_{t}},\Sigma_{\epsilon ,y_{t}}\right)p\left(y_{t}^{i}|p_{1:J}\right)p\left(p_{i:J};\alpha_{t}\right)p\left({\bar{z}}_{y_{t}};\mu_{y_{t},t},\Lambda_{y_{t},t}^{-1}\right) \end{array}$$

(A5) $$\begin{array}{cc} & =N\left(\mu_{t+1},\Sigma_{t+1}\right)\mathrm{Dir}\left(\alpha_{t+1}\right), \end{array}$$

where $N_{t}$ is the number of data points seen during update *t*. Concentrating on the Categorical-Dirichlet conjugacy:

(A6) $$\begin{array}{cc} \mathrm{Dir}(\alpha_{t+1}) & \propto p\left(p_{1:J};\alpha_{t}\right)\prod_{i=1}^{N_{t}}p\left(y_{t}^{i};p_{i:J}\right) \end{array}$$

(A7) $$\begin{array}{cc} \; & \propto \prod_{j=1}^{J}p_{j}^{\alpha_{j}-1}\prod_{i=1}^{N_{t}}\prod_{j=1}^{J}p_{j}^{I(y_{t}^{i}=j)} \end{array}$$

(A8) $$\begin{array}{cc} \; & =\prod_{j=1}^{J}p_{j}^{\alpha_{j}-1+\sum_{i=1}^{N_{t}}I\left(y_{t}^{i}=j\right)}. \end{array}$$

Thus:

(A9) $$\begin{array}{c} \alpha_{t+1,j}=\alpha_{t,j}+\sum_{i=1}^{N_{t}}I\left(y_{t}^{i}=j\right). \end{array}$$

Moreover, due to Gaussian-Gaussian conjugacy, then the posterior for the Gaussian prototype of the embedding for each class is:

(A10) $$\begin{array}{cc} N(\mu_{t+1},\Lambda_{t+1}) & \propto \prod_{i=1}^{N_{t}}N\left(z_{t}^{i}|y_{t}^{i};{\bar{z}}_{y_{t}},\Sigma_{\epsilon }\right)N\left({\bar{z}}_{y_{t}};\mu_{y_{t},t},\Lambda_{y_{t}}^{-1}\right) \end{array}$$

(A11) $$\begin{array}{cc} \; & =\prod_{y_{t}\in \left\{1,\ldots ,J\right\}}N\left(z_{y_{t}}|y_{t};{\bar{z}}_{y_{t}},\frac{1}{N_{y_{t}}}\Sigma_{\epsilon }\right)N\left({\bar{z}}_{y_{t}};\mu_{y_{t+1}},\Lambda_{y_{t}}^{-1}\right) \end{array}$$

(A12) $$\begin{array}{cc} \; & =\prod_{y_{t}\in \left\{1,\ldots ,J\right\}}N\left({\bar{z}}_{y_{t}};\mu_{t+1},\Lambda_{y_{t+1}}^{-1}\right), \end{array}$$

where $N_{y_{t}}$ is the number of points of class $y_{t}$ from the set of all classes C={1,…,J}. The update equations for the mean and variance of the posterior are:

(A13) $$\begin{array}{cc} \Lambda_{y_{t+1}} & =\Lambda_{y_{t}}+N_{y_{t}}\Sigma_{\epsilon }^{-1},\;\forall y_{t}\in C_{t} \end{array}$$

(A14) $$\begin{array}{cc} \Lambda_{y_{t+1}}\mu_{y_{t+1}} & =N_{y_{t}}\Sigma_{\epsilon }^{-1}{\bar{z}}_{y_{t}}+\Lambda_{y_{t}}\mu_{y_{t}},\;\forall y_{t}\in C_{t}. \end{array}$$

### Appendix D.3. ProtoCL Objective

The posterior predictive distribution we want to optimize is:

(A15) p(z,y)=∫p(z,y|θ;η)p(θ;η)dθ,

where p(θ) denotes the distributions over class probabilities ${\left\{p_{j}\right\}}_{j=1}^{J}$ and mean embeddings ${\left\{{\bar{z}}_{y_{j}}\right\}}_{j=1}^{J}$,

(A16) $$\begin{array}{cc} p(z,y) & =\int \prod_{i=1}^{N_{t}}p\left(z_{it}|y_{it};{\bar{z}}_{y_{t}},\Sigma_{\epsilon }\right)p\left(y_{it}|p_{1:J}\right)p\left(p_{1:J};\alpha_{t}\right)p\left({\bar{z}}_{y_{t}};\mu_{y_{t},t},\Lambda_{y_{t},t}^{-1}\right)dp_{1:J}d{\bar{z}}_{y_{t}} \end{array}$$

(A17) $$\begin{array}{cc} \; & =\int \prod_{i=1}^{N_{t}}p\left(z_{it}|y_{it};z_{y_{t}},\Sigma_{\epsilon }\right)p\left({\bar{z}}_{y_{t}};\mu_{y_{t},t},\Lambda_{y_{t},t}^{-1}\right)d{\bar{z}}_{y_{t}}\underset{\prod_{i}p\left(y_{i}\right)=p\left(y\right)}{\underbrace{\int \prod_{i=1}^{N_{t}}p\left(y_{it}|p_{1:J}\right)p\left(p_{1:J};\alpha_{t}\right)dp_{1:J}}} \end{array}$$

(A18) $$\begin{array}{cc} \; & =p\left(y\right)\prod_{i=1}^{N_{t}}Z_{i}^{-1}\int N\left({\bar{z}}_{y_{it}};c,C\right)d{\bar{z}}_{y_{t}} \end{array}$$

(A19) $$\begin{array}{cc} \; & =p\left(y\right)\prod_{i=1}^{N_{t}}N\left(z_{it}|y_{it};\mu_{y_{t},t},\Sigma_{\epsilon }+\Lambda_{y_{t},t}^{-1}\right). \end{array}$$

where in Equation (A18) we use §8.1.8 in [54]. The term p(y) is:

(A20) $$\begin{array}{cc} p(y) & =\int p\left(y|p_{1:J}\right)p\left(p_{1:J};\alpha_{t}\right)dp_{1:J} \end{array}$$

(A21) $$\begin{array}{cc} \; & =\int p_{y}\frac{\Gamma (\sum_{j=1}^{J}\alpha_{j})}{\prod_{j=1}^{J}\Gamma \left(\alpha_{j}\right)}\prod_{j=1}^{J}p_{j}^{\alpha_{j}-1}dp_{1:J} \end{array}$$

(A22) $$\begin{array}{cc} \; & =\frac{\Gamma (\sum_{j=1}^{J}\alpha_{j})}{\prod_{j=1}^{J}\Gamma \left(\alpha_{j}\right)}\int \prod_{j=1}^{J}p_{j}^{I\left(y=j\right)+\alpha_{j}-1}dp_{1:J} \end{array}$$

(A23) $$\begin{array}{cc} \; & =\frac{\Gamma (\sum_{j=1}^{J}\alpha_{j})}{\prod_{j=1}^{J}\Gamma \left(\alpha_{j}\right)}\frac{\prod_{j=1}^{J}\Gamma \left(I\left(y=j\right)+\alpha_{j}\right)}{\Gamma (1+\sum_{j=1}^{J}\alpha_{j})} \end{array}$$

(A24) =Γ(∑j=1Jαj)∏j=1JΓ(αj)∏j=1JΓ(I(y=j)+αj)∑j=1JαjΓ(∑j=1Jαj)

(A25) $$\begin{array}{cc} \; & =\frac{\prod_{j=1,j\neq y}^{J}\Gamma \left(\alpha_{j}\right)}{\prod_{j=1}^{J}\Gamma \left(\alpha_{j}\right)}\frac{\Gamma (1+\alpha_{y})}{\sum_{j=1}^{J}\alpha_{j}} \end{array}$$

(A26) $$\begin{array}{cc} \; & =\frac{\prod_{j=1,j\neq y}^{J}\Gamma \left(\alpha_{j}\right)}{\prod_{j=1}^{J}\Gamma \left(\alpha_{j}\right)}\frac{\alpha_{y}\Gamma \left(\alpha_{y}\right)}{\sum_{j=1}^{J}\alpha_{j}} \end{array}$$

(A27) $$\begin{array}{cc} \; & =\frac{\alpha_{y}}{\sum_{j=1}^{J}\alpha_{j}}, \end{array}$$

where we use the identity Γ(n+1)=nΓ(n).

### Appendix D.4. Predictions

To make a prediction for a test point $x^{*}$:

(A28) $$\begin{array}{cc} p(y^{*}=j|x^{*},x_{1:t},y_{1:t}) & =p(y^{*}=j|z^{*},\theta_{t}) \end{array}$$

(A29) $$\begin{array}{cc} \; & =\frac{p\left(z^{*}|y^{*}=j,\theta_{t}\right)p\left(y^{*}=j|\theta_{t}\right)}{\sum_{i}p\left(z^{*}|y^{*}=i,\eta_{t}\right)p\left(y^{*}=i|\theta_{t}\right)} \end{array}$$

(A30) $$\begin{array}{cc} \; & =\frac{p(y^{*}=j,z^{*}|\theta_{t})}{\sum_{i}p\left(y=i,z^{*}|\theta_{t}\right)}, \end{array}$$

where $\theta_{t}$ are sufficient statistics for $\left(x_{1:t},y_{1:t}\right)$.

**Preventing forgetting.** As we wish to retain the task-specific prototypes, at the end of learning a task $T_{t}$ we take a small subset of the data as a memory to ensure that posterior parameters and prototypes do not drift, see Algorithm 1.

### Appendix D.5. Experimental Setup

The prototype variance, $\Sigma_{\epsilon }$ is set to a diagonal matrix with the variances of each prototype set to 0.05. The prototype prior precisions, $\Lambda_{yt}$, are also diagonals and initialized randomly and exponentiated to ensure a positive semi-definite covariance for the sequential updates. The parameters $\alpha_{j}\;\forall j$ are set to 0.78, which was found by random search over the validation set on MNIST. We also allow $\alpha_{j}$ to be learned in the gradient update step in addition to the sequential update step (lines 4 and 5 Algorithm 1), see Figure A7 to see the evolution of the $\alpha_{j}$ or all classes *j* over the course of learning Split-MNIST.

For the Split-MNIST and Split-FMNIST benchmarks, we use an NN with two layers of size 200 and trained for 50 epochs with an Adam optimizer. We perform a grid-search over learning rates, dropout rates, and weight decay coefficients. The embedding dimension is set to 128. For the Split-CIFAR10 and Split-CIFAR100 benchmarks, we use the same network as Pan et al. [22], which consists of four convolution layers and two linear layers. We train the networks for 80 epochs for each task with the Adam optimizer with a learning rate of $1\times 10^{-3}$. The embedding dimension is set to 32. All experiments are run on a single GPU NVIDIA RTX 3090.

## Appendix E. Sequential Bayesian Estimation as Bayesian Neural Network Optimization

We shall consider inference in the graphical model depicted in Figure A8. The aim is to infer the optimal BNN weights, $\theta_{t}^{*}$ at time *t* given observations and the previous BNN weights. We assume a Gaussian posterior over weights with full covariance; hence, we model interactions between all weights. We shall consider the online setting where we see one data point $\left(x_{t},y_{t}\right)$ at a time and we will make no assumption as to whether the data comes from the same task or different tasks over the course of learning.

We set up the problem of sequential Bayesian inference as a filtering problem and we leverage the work of Aitchison [31], which casts NN optimization as Bayesian sequential inference. We make the reasonable assumption that the distribution over weights is a Gaussian with full covariance. Since reasoning about the full covariance matrix of a BNN is intractable, we instead consider the *i*-th parameter and reason about the dynamics of the optimal estimates $\theta_{it}^{*}$ as a function of all the other parameters $\theta_{-it}$. Each weight is functionally dependent on all others. If we had access to the full covariance of the parameters, then we could reason about the unknown optimal weights $\theta_{it}^{*}$ given the values of all the other weights $\theta_{-it}$. However, since we do not have access to the full covariance, another approach is to reason about the dynamics of $\theta_{it}^{*}$ given the dynamics of $\theta_{-it}$ and assume that the values of the weights are close to those of the previous timestep [32] and so we cast the problem as a dynamical system.

Consider a quadratic loss of the form:

(A31) $$\begin{array}{c} L\left(x_{t},y_{t};\theta \right)=L_{t}\left(\theta \right)=-\frac{1}{2}\theta^{⊤}H\theta +z_{t}^{⊤}\theta , \end{array}$$

which we can arrive at by simple Taylor expansion, where H is the Hessian which is assumed to be constant across data points but not across the parameters θ. If the BNN output takes the form $f(x_{t};\theta )$, then the derivative evaluated at $\theta_{t}$ is $z_{t}=\frac{\partial L(x_{t},y_{t};\theta )}{\partial \theta }{|}_{\theta =\theta_{t}}$. To construct the dynamical equations for our weights, consider the gradient for a single datapoint:

(A32) $$\begin{array}{c} \frac{\partial L_{t}\left(\theta \right)}{\partial \theta }=-H\theta +z_{t}. \end{array}$$

If we consider the gradient for the *i*-th weight while all other parameters are set to their current estimate:

(A33) $$\begin{array}{c} \frac{\partial L(\theta_{i},\theta_{-i})}{\partial \theta_{i}}=-H_{ii}\theta_{it}-H_{-ii}^{⊤}\theta_{-it}+z_{ti}. \end{array}$$

When the gradient is set to zero we recover the optimal value for $\theta_{it}$, denoted as $\theta_{it}^{*}$:

(A34) $$\begin{array}{c} \theta_{it}^{*}=-\frac{1}{H_{ii}}H_{-ii}^{⊤}\theta_{-it}. \end{array}$$

since $z_{ti}=0$ at the modes. The equation above shows us that the dynamics of the optimal weight $\theta_{it}^{*}$ is dependent on all the other current values of the parameters $\theta_{-it}$. That is, the dynamics of $\theta_{it}^{*}$ are governed by the dynamics of the weights $\theta_{-it}$. The dynamics of $\theta_{-it}$ are a complex stochastic process dependent on many different variables. Since reasoning about the dynamics is intractable, we instead assume a discretized Ornstein–Uhlenbeck process for the weights $\theta_{-it}$ with reversion speed $\vartheta \in R_{+}$ and noise variance $\eta_{-i}^{2}$:

(A35) $$\begin{array}{c} p\left(\theta_{-i,t+1}|\theta_{-i,t}\right)=N\left(\left(1-\vartheta \right)\theta_{-it},\eta_{-i}^{2}\right), \end{array}$$

this implies that the dynamics for the optimal weight are defined by

(A36) $$\begin{array}{c} p\left(\theta_{i,t+1}^{*}|\theta_{i,t}^{*}\right)=N\left(\left(1-\vartheta \right)\theta_{it}^{*},\eta^{2}\right), \end{array}$$

where $\eta^{2}=\eta_{-i}^{2}H_{-ii}^{⊤}H_{-ii}$. This same assumption is made in Aitchison [31]. This assumes a parsimonious model of the dynamics. Together with our likelihood:

(A37) $$\begin{array}{c} p\left(y_{t}|x_{t};\theta_{t}^{*}\right)=N\left(y_{t};f\left(x_{t};\theta_{t}^{*}\right),\sigma^{2}\right) \end{array}$$

where f(·,θ) is a neural network prediction with weights θ, we can now define a linear dynamical system for the optimal weight $\theta_{i}^{*}$ by linearizing the Bayesian NN [32] and by using the transition dynamics in Equation (A36). Thus, we are able to infer the posterior distribution over the optimal weights using Kalman filter-like updates [25]. As the dynamics and likelihood are Gaussian, then the prior and posterior are also Gaussian, for ease of notation we drop the index *i* such that $\theta_{it}^{*}=\theta_{t}^{*}$:

(A38) $$\begin{array}{cc} p(\theta_{t}^{*}|{\left(x,y\right)}_{t-1},\ldots ,{\left(x,y\right)}_{1}) & =N(\mu_{t,\mathrm{prior}},\sigma_{t,\mathrm{prior}}^{2}) \end{array}$$

(A39) $$\begin{array}{cc} p(\theta_{t}^{*}|{\left(x,y\right)}_{t},\ldots ,{\left(x,y\right)}_{1}) & =N(\mu_{t,\mathrm{post}},\sigma_{t,\mathrm{post}}^{2}) \end{array}$$

By using the transition dynamics and the prior we can obtain closed-form updates:

(A40) $$\begin{array}{cc} p(\theta_{t}^{*}|{\left(x,y\right)}_{t-1},\ldots ,{\left(x,y\right)}_{1}) & =\int p\left(\theta_{t}^{*}|\theta_{t-1}^{*}\right)p\left(\theta_{t-1}^{*}|{\left(x,y\right)}_{t-1},\ldots ,{\left(x,y\right)}_{1}\right)d\theta_{t-1}^{*} \end{array}$$

(A41) $$\begin{array}{cc} N(\theta_{t}^{*};\mu_{t,\mathrm{prior}},\sigma_{t,\mathrm{prior}}^{2}) & =\int N\left(\theta_{t}^{*};\left(1-\vartheta \right)\theta_{t-1}^{*},\eta^{2}\right)N\left(\theta_{t-1}^{*};\mu_{t-1,\mathrm{post}},\sigma_{t-1,\mathrm{post}}^{2}\right)d\theta_{t-1}^{*}. \end{array}$$

Integrating out $\theta_{t-1}^{*}$ we can obtain updates for the prior for the next timestep as follows:

(A42) $$\begin{array}{cc} \mu_{t,\mathrm{prior}} & =\left(1-\vartheta \right)\mu_{t-1,post} \end{array}$$

(A43) $$\begin{array}{cc} \sigma_{t,\mathrm{prior}}^{2} & =\eta^{2}+{\left(1-\vartheta \right)}^{-2}\sigma_{t-1,\mathrm{post}}^{2}. \end{array}$$

The updates for obtaining our posterior parameters: $\mu_{t,\mathrm{post}}$ and $\sigma_{t,\mathrm{post}}^{2}$, comes from applying Bayes’ theorem:

(A44) $$\begin{array}{cc} \log N(\theta_{t}^{*};\mu_{t,\mathrm{post}},\sigma_{t,\mathrm{post}}^{2}) & \propto \log N\left(y_{t};f\left(x_{t};\theta_{t}^{*}\right),\sigma^{2}\right)+\log N\left(\theta_{t}^{*};\mu_{t,\mathrm{prior}},\sigma_{t,\mathrm{prior}}^{2}\right), \end{array}$$

by linearizing our Bayesian NN such that $f\left(x_{t},\theta_{0}\right)\approx f\left(x_{t},\theta_{0}\right)+\frac{\partial f(x_{t};\theta_{t}^{*})}{\partial \theta_{t}^{*}}\left(\theta_{t}^{*}-\theta_{0}\right)$ and by substituting into Equation (A44) we obtain our update equation for the posterior of the mean of our BNN parameters:

(A45) $$\begin{array}{cc} -\frac{1}{2\sigma_{t,\mathrm{post}}^{2}}{\left(\theta_{t}^{*}-\mu_{t,\mathrm{post}}\right)}^{2} & =-\frac{1}{2\sigma^{2}}{\left(y-g\left(x_{t}\right)\theta_{t}^{*}\right)}^{2}-\frac{1}{2\sigma_{t,\mathrm{prior}}^{2}}{\left(\theta_{t}^{*}-\mu_{t,\mathrm{prior}}\right)}^{2} \end{array}$$

(A46) $$\begin{array}{cc} \mu_{t,\mathrm{post}} & =\sigma_{t,\mathrm{post}}^{2}\left(\frac{\mu_{t,\mathrm{prior}}}{\sigma_{t,\mathrm{prior}}^{2}}+\frac{y}{\sigma^{2}}g\left(x_{t}\right)\right), \end{array}$$

where $g\left(x_{t}\right)=\frac{\partial f(x_{t};\theta_{t}^{*})}{\partial \theta_{t}^{*}}$, and the update equation for the variance of the Gaussian posterior is:

(A47) $$\begin{array}{cc} \frac{1}{\sigma_{t,\mathrm{post}}^{2}} & =\frac{g{\left(x_{t}\right)}^{2}}{\sigma^{2}}+\frac{1}{\sigma_{t,\mathrm{prior}}^{2}}. \end{array}$$

From our updated equations, Equation (A46) and Equation (A47), we can notice that the posterior mean depends linearly on the prior and an additional data dependent term. These equations are similar to the filtering example in sec:misspecification. Therefore, under certain assumptions above, a BNN should behave similarly. If there exists a task data imbalance, then the data term will dominate the prior term in Equation (A46) and could lead to forgetting of previous tasks.
